# Metabolic Transcriptional Activation in Ulcerative Colitis Identified Through scRNA-seq Analysis

**DOI:** 10.3390/genes15111412

**Published:** 2024-10-31

**Authors:** Christophe Desterke, Yuanji Fu, Raquel Francés, Jorge Mata-Garrido

**Affiliations:** 1 Faculté de Médecine du Kremlin Bicêtre, University Paris-Sud, Université Paris-Saclay, 94270 Le Kremlin-Bicêtre, France; christophe.desterke@inserm.fr; 2 INSERM, CNRS, Institut Necker Enfants Malades, Université Paris Cité, 75015 Paris, France; yuanji.fu@inserm.fr; 3 Energy & Memory, Brain Plasticity Unit, CNRS, ESPCI Paris, PSL Research University, 75006 Paris, France; raquel.frances@espci.fr; 4 INSERM U993, Unité Organisation Nucléaire et Oncogenèse, Institut Pasteur, Université Paris Cité, 75006 Paris, France

**Keywords:** ulcerative colitis, transcriptome, enzyme, metabolism

## Abstract

Background: Ulcerative colitis is a chronic inflammatory disease affecting the colon. During chronic inflammation of epithelial cells, lipid metabolism via pro-inflammatory eicosanoids is known to modify the immune response. Methods: Starting from the Mammalian Metabolic Database, the expression of metabolic enzymes was investigated in two independent cohorts from transcriptome datasets GSE38713 and GSE11223, which analyzed ulcerative colitis tissue samples from the digestive tract. Results: In the first cohort, 145 differentially expressed enzymes were identified as significantly regulated between ulcerative colitis tissues and normal controls. Overexpressed enzymes were selected to tune an Elastic Net model in the second cohort. Using the best parameters, the model achieved a prediction accuracy for ulcerative colitis with an area under the curve (AUC) of 0.79. Twenty-two metabolic enzymes were found to be commonly overexpressed in both independent cohorts, with decreasing Elastic Net predictive coefficients as follows: LIPG (3.98), PSAT1 (3.69), PGM3 (2.74), CD38 (2.28), BLVRA (1.99), CBR3 (1.94), NT5DC2 (1.76), PHGDH (1.71), GPX7 (1.58), CASP1 (1.56), ASRGL1 (1.4), SOD3 (1.25), CHST2 (0.965), CHST11 (0.95), KYNU (0.94), PLAG2G7 (0.92), SRM (0.87), PTGS2 (0.80), LPIN1 (0.47), ME1 (0.31), PTGDS (0.14), and ADA (0.13). Functional enrichment analysis using the Kyoto Encyclopedia of Genes and Genomes (KEGG) database highlighted the main implications of these enzymes in cysteine and methionine metabolism (adjusted *p*-value = 0.01), arachidonic acid and prostaglandin metabolism (adjusted *p*-value = 0.01), and carbon metabolism (adjusted *p*-value = 0.04). A metabolic score based on the transcriptional activation of the validated twenty-two enzymes was found to be significantly greater in Ulcerative colitis samples compared to healthy donor samples (*p*-value = 1.52 × 10^−8^). Conclusions: A metabolic expression score was established and reflects the implications of heterogeneous metabolic pathway deregulations in the digestive tract of patients with ulcerative colitis.

## 1. Introduction

Ulcerative colitis (UC) is a chronic inflammatory bowel disease (IBD) that primarily affects the colon and rectum, leading to mucosal ulceration and persistent inflammation. The disease has a significant prevalence worldwide, affecting approximately 0.5 to 1 million people in the United States alone [1,2]. UC typically presents in episodes of acute exacerbations followed by periods of remission, with symptoms including abdominal pain, diarrhea (often bloody), and rectal bleeding [3]. The pathophysiology of UC is complex and multifactorial, involving genetic susceptibility, environmental factors, and dysregulation of the immune system [4,5]. As a result of these contributing factors, patients with UC are at an increased risk of complications, such as colorectal cancer and severe colonic damage.

A hallmark of UC is the excessive inflammatory response observed in the intestinal mucosa [6]. The inflammation is characterized by an infiltration of immune cells, including T lymphocytes, macrophages, and neutrophils, which contribute to the pathological state of the disease [7]. The immune response in UC is triggered by various factors, including microbial antigens, dietary components, and host-derived signals. This aberrant immune activation leads to the release of pro-inflammatory cytokines and chemokines, perpetuating inflammation and resulting in tissue damage. In particular, the balance between pro-inflammatory and anti-inflammatory mediators is crucial for maintaining intestinal homeostasis [4,5,6,7]. Dysregulation of this balance can lead to sustained inflammation and the development of chronic inflammatory conditions such as UC. In this context, the PD-L1/PD-1 immune checkpoint pathway plays a crucial role in modulating immune responses in the intestinal mucosa [8,9]. PD-L1 is often upregulated in response to the chronic inflammation observed in UC, particularly in immune and epithelial cells, as a compensatory mechanism to limit excessive T-cell activation [10]. However, despite this upregulation, the immune checkpoint function may become ineffective, either due to overwhelming inflammatory stimuli or T-cell exhaustion, contributing to the uncontrolled immune activation that perpetuates the disease [8,9,10]. This imbalance highlights the potential importance of the PD-L1 pathway in UC pathophysiology, and its targeting may represent a novel therapeutic approach to restore immune regulation and reduce mucosal damage. However, more research is needed to fully assess the therapeutic potential and safety of modulating the PD-1–PD-L1 axis in UC.

Recent studies have underscored the critical role of metabolism in the pathogenesis of UC, linking metabolic alterations to the disease process [11]. The colon harbors a diverse and complex microbiota that plays a pivotal role in maintaining gut health and homeostasis [12]. The interaction between the gut microbiota and host metabolism is vital for various physiological processes, including nutrient absorption, immune modulation, and the production of bioactive metabolites. Metabolites produced by gut bacteria, such as short-chain fatty acids (SCFAs), are essential for maintaining intestinal barrier integrity and regulating immune responses [13,14]. In UC, changes in the composition and function of the gut microbiota have been observed, leading to dysbiosis, which may further exacerbate inflammation and tissue injury [12,13,14]. This relationship between gut microbiota and IBD has garnered significant attention in recent years, raising the question of whether this association is one of causation or correlation. While it is well established that dysbiosis is frequently observed in patients with IBD, the exact nature of this relationship remains complex [12,13,14]. On the one hand, changes in microbial diversity and abundance may contribute to the pathogenesis of IBD by disrupting immune homeostasis and promoting inflammatory pathways [15]. Conversely, the inflammatory environment characteristic of IBD could also alter the gut microbiota composition, suggesting a bidirectional relationship [15]. Recent studies utilizing advanced metagenomic techniques and animal models have begun to elucidate specific microbial taxa that may play causal roles in IBD [16]. However, further research is needed to clarify the mechanisms underlying these interactions and determine whether restoring a healthy microbiota could serve as a therapeutic strategy for managing IBD. Tissular alterations in lipid, carbohydrate, and amino acid metabolism have also been implicated in the disease’s progression [8]. For instance, the metabolism of eicosanoids, which are bioactive lipids derived from fatty acids, plays a crucial role in mediating inflammatory responses [17,18,19]. In UC, the overproduction of pro-inflammatory eicosanoids can contribute to the amplification of the inflammatory cascade, leading to exacerbated symptoms and disease severity [17,18,19]. Eicosanoids are bioactive lipids derived from the metabolism of arachidonic acid and play a crucial role in the inflammatory processes associated with ulcerative colitis (UC) [20]. In UC, the excessive production of pro-inflammatory eicosanoids, such as prostaglandins and leukotrienes, contributes significantly to the pathology of the disease [21]. These mediators are synthesized by various enzymes, including cyclooxygenases (COX) and lipoxygenases (LOX), and are released in response to immune stimuli [21]. In contrast, certain eicosanoids, such as lipoxins and resolvins, have been shown to play protective roles by promoting inflammation resolution and tissue repair [22,23]. Therefore, the balance between pro-inflammatory and anti-inflammatory eicosanoids is critical in determining the severity and progression of UC, highlighting their potential as therapeutic targets in managing this chronic inflammatory disease.

This study aims to elucidate the connection between metabolism transcriptional program dysregulation and the inflammatory processes in ulcerative colitis by investigating the expression of metabolic enzymes in colonic tissues. We hypothesize that specific metabolic pathways are significantly altered in ulcerative colitis patients, contributing to the disease’s inflammatory response and progression. By analyzing transcriptomic data from ulcerative colitis patients, we seek to identify key metabolic pathways involved in the disease’s progression. Our findings will contribute to a better understanding of the metabolic alterations that accompany UC and their potential implications for therapeutic intervention. Understanding these metabolic pathways may pave the way for novel strategies targeting the metabolic disturbances observed in ulcerative colitis, ultimately improving patient outcomes.

## 2. Materials and Methods

### 2.1. PICO Statement

P-population of patients: Ulcerative colitis transcriptome cohorts include patients with diverse active disease statuses and disease extents in the first cohort (Table 1), as well as variations in tissue and pathology localization, family history, UC staging score (UCSS), progression, smoking status, inflammatory status in the second cohort (Table 2).

I-intervention: Supervised analysis of the metabolic transcriptional program within the complete transcriptome of their pathological tissue.

C-comparison: Pathological tissues were directly compared to their relative healthy tissue control.

O-outcome: Binary outcome of the disease status.

### 2.2. Public Datasets

#### 2.2.1. Training Transcriptome Dataset of Ulcerative Colitis Tissues

The GSE38713 [24] training dataset was downloaded using the GEOquery R-Bioconductor package version 2.70.0 [25] from the Gene Expression Omnibus website [26], which included ulcerative colitis patients and non-inflammatory controls. Inclusion criteria for UC patients were age between 18 and 65, diagnosis of UC established at least 6 months before inclusion, and exclusion of concomitant infection. A total of 43 biopsies were analyzed: 13 healthy controls, 8 inactive UC cases, 7 non-involved active UC cases, and 15 involved active UC cases.

#### 2.2.2. Validation Transcriptome Dataset of Ulcerative Colitis Tissues

The GSE11223 [27] validation dataset was downloaded from the Gene Expression Omnibus website [26] at the following address: https://www.ncbi.nlm.nih.gov/geo/query/acc.cgi?acc=GSE11223 (accessed on 23 September 2024). It was then annotated using the corresponding technological platform: Agilent-012391 Whole Human Genome Oligo Microarray G4112A available at the following address: https://www.ncbi.nlm.nih.gov/geo/query/acc.cgi?acc=GPL1708 (accessed on 23 September 2024). This dataset is composed of transcriptome experiments performed on 73 intestinal controls and 129 ulcerative colitis samples. Samples of controls were collected from distinct regions of the digestive tract: 17 from the ascending colon, 23 from the descending colon, 27 from the sigmoid colon, and 6 from the terminal ileum.

### 2.3. Mammalian Metabolic Transcriptional Program

The Mammalian Metabolic Enzyme Database [28] was downloaded from the following web address: https://esbl.nhlbi.nih.gov/Databases/KSBP2/Targets/Lists/MetabolicEnzymes/MetabolicEnzymeDatabase.html (accessed on 23 September 2024). It was then annotated using the Ensembl Biomart database version 110 [29] through the geneconverter R-package, accessible at the following address: https://github.com/cdesterke/geneconverter (accessed on 23 September 2024).

### 2.4. Transcriptome Analyses

Transcriptome analyses were performed in the R software environment, version 4.3.3. Differential expression analysis was performed using the transpipe14 R-package, which implements the limma algorithm [30] and is available at the following address: https://github.com/cdesterke/transpipe14 (accessed on 23 September 2024) [31]. Expression heatmaps and unsupervised clustering (using Euclidean distances and Ward.D2 methods) were generated with the support of the heatmap R package, version 1.0.12.

### 2.5. Elastic Net Machine Learning Tuning

Enzymes found to be overexpressed (log2 fold change ≥ 1) in ulcerative colitis samples from the training cohort (GSE38713) were selected for validation in the dataset (GSE11223) to be tested as covariates in a binomial Elastic Net model testing UC status: negative/positive. After splitting the data into training and validation sets (0.7/0.3 ratio), the Elastic Net model (UC status as the binary outcome) was tuned on Alpha and lambda parameters using the caret R package, version 6.0-94 [32]. The final Elastic Net model was fit with the best Alpha parameter (Alpha = 0.4), using the glmnet R package version 4.1-8 [33,34].

### 2.6. Elastic Net (Enet) Expression Score Computing

For the enzymes confirmed with a positive coefficient in the Elastic Net model, an Elastic Net expression score was computed on the validation dataset GSE11223 according to the following formula:

enet.score = Σ expression × coefficient

where “expression” represents the expression level of each enzyme and “coefficient” represents the Elastic Net coefficient for each enzyme. The optimal threshold of the net.score to predict ulcerative colitis diagnosis was computed using the cutpointr R package version 1.1.2 [35]. ROC curve analyses were performed using the pROC R package version [36]. Mosaic plots and chi-square tests were performed using the vcd R package version 1.4-12 [37].

### 2.7. KEGG Enrichment Network

Using the twenty-two enet enzyme signature, Kyoto Encyclopedia of Genes and Genomes (KEGG) [38] functional enrichment was performed using the ClusterProfiler R-Bioconductor package version 4.10.1 [39]. Individual KEGG-enriched pathways were visualized using the pathview R package version 1.42.0 [40]. The KEGG enrichment network was generated using the Cytoscape standalone application version 3.9.1 [41].

## 3. Results

### 3.1. Metabolic Enzymes Regulated During Ulcerative Colitis

**Figure 1:**
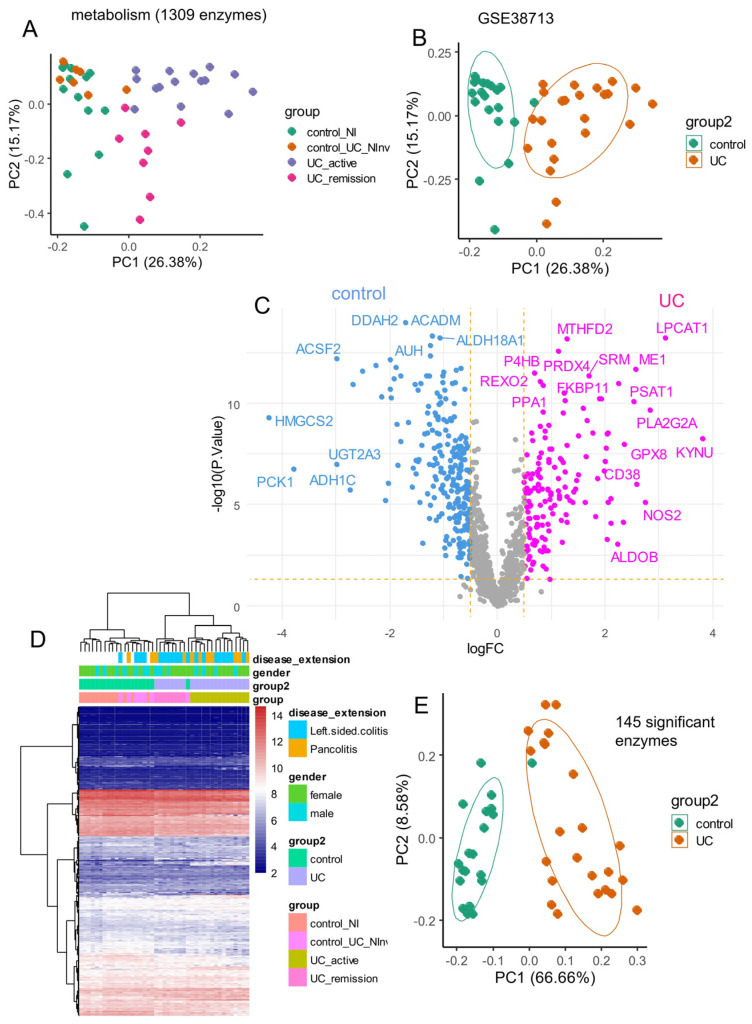
Differentially expressed enzymes between ulcerative colitis and control tissue in training cohort: dataset GSE38713. (**A**) Principal component analysis performed on the expression of 1309 enzymes and stratified by subtypes of disease stages: UC_Ninv: non-involved active UC; UC_active, UC_remission, and control; (**B**) Principal component analysis on the expression of 1309 enzymes and stratified between UC (ulcerative colitis) and non-inflammatory control tissues; (**C**) Volcano plot of differentially expressed enzymes between ulcerative colitis and non-inflammatory control; (**D**) Unsupervised clustering (Euclidean distances) and expression heatmap performed with 145 differential expressed enzymes; (**E**) Principal component analysis performed on the expression of 145 differential expressed enzymes and stratified between UC and controls.

Using enzymes collected from the Mammalian Metabolic Database [28], an unsupervised principal component analysis (PCA) was performed on the training dataset GSE38713, which quantified 1309 of them (Figure 1A). This dataset comprised 23 samples of the ulcerative colitis group (UC in remission and active UC) versus 20 controls (non-inflammatory colon controls and UC tissue that was non-involved or not affected by the disease) without sampling bias for gender and age (Table 1). This PCA analysis revealed a good stratification of controls and ulcerative colitis samples along the first principal axis, which accounted for 26.38% of the variance (Figure 1B). In the secondary step, differential expression analysis was performed between the supervised classes: control and ulcerative colitis (Figure 1C). Subsequently, a differential expression analysis between controls and ulcerative colitis samples using the LIMMA algorithm was performed on metabolic enzyme expression, identifying 145 significantly differentially expressed enzymes between the groups (Supplemental Table S1, DOI: 10.6084/m9.figshare.27088645). Unsupervised clustering based on Euclidean distances using these enzymes enabled effective sample stratification, independent of gender and disease extent (Figure 1D). Clustering of samples from Figure 1D identified two main branches in the tree: one on the left comprising a majority of controls, including control samples and UC tissues non-involved by the disease, and one major branch on the right comprising UC pathological tissues. The right branch was also cut into two sub-branches: the left sub-cluster of the right main cluster near controls assembled UC remission and UC non-inflammatory samples together, while the right sub-cluster comprised only active UC samples. These results suggest that the 145-metabolism signature is efficient for discriminating UC samples from controls but also for discriminating active from non-active disease (Figure 1D). When PCA was limited to these 145 enzymes, the variance explained by the first principal axis increased to 66.66% (Figure 1E). These results suggest a distinct metabolic expression program in the digestive tracts of ulcerative colitis patients compared to non-inflammatory controls.

### 3.2. Elastic Net Validation of a 22-Enzyme Signature Activated in an Independent Cohort of Ulcerative Colitis

**Figure 2:**
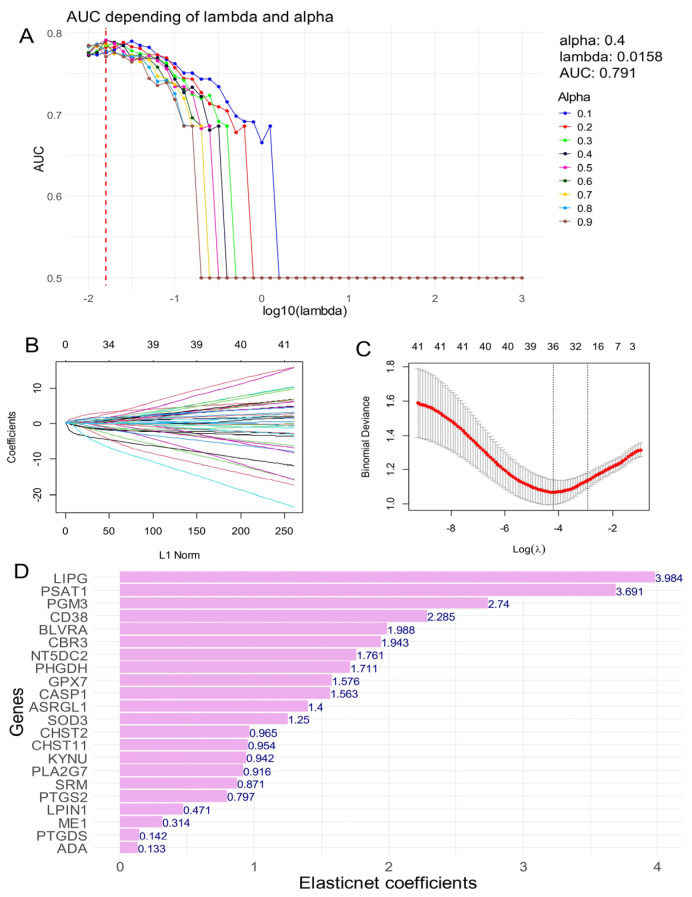
Validation of activated enzymes in ulcerative colitis by Elastic Net tuning on the independent transcriptome cohort: dataset GSE11223. (**A**) Tuning of Alpha and lambda parameters during Elastic Net regularization for the binary outcome: Ulcerative colitis status (negative/positive), best parameters and best area under curve (AUC) are indicated; (**B**) Elastic Net machine learning fit with best Alpha parameter fixed at 0.4; (**C**) Elastic Net coefficient of variation on the lambda parameter for Alpha fixed at 0.4; (**D**) Bar plot of positive Elastic Net coefficients for the optimal Elastic Net model to predict ulcerative colitis status.

From the differentially expressed enzymes identified in the training cohort (GSE38713; Supplemental Table S1), we selected overexpressed enzymes with a log2-fold change of ≥1 and an adjusted *p*-value of ≤0.05 for validation in the GSE11223 cohort using machine learning with Elastic Net regularization (Figure 2). The GSE11223 cohort was split into two subsets (70% training and 30% testing) for model development. After defining a sequence for the lambda and Alpha parameters, models were constructed using the larger training set, and the area under the curve (AUC) was estimated on the smaller test set (Figure 2A). The optimal AUC of 0.79 was achieved with an Alpha parameter set to 0.4 (Figure 2A). A secondary Elastic Net model was then built with the Alpha parameter fixed at 0.4 (Figure 2B), and the coefficient of variation of the model on the lambda parameter confirmed the identification of 22 enzymes with positive Elastic Net coefficients in the validation cohort, indicating their potential to predict a positive status for ulcerative colitis (Figure 2C). The results are illustrated in Figure 2D.

### 3.3. Enzyme Elastic Net Expression (Enet) Score Is a Significant Marker to Predict Ulcerative Colitis in Colon Tissue

**Figure 3:**
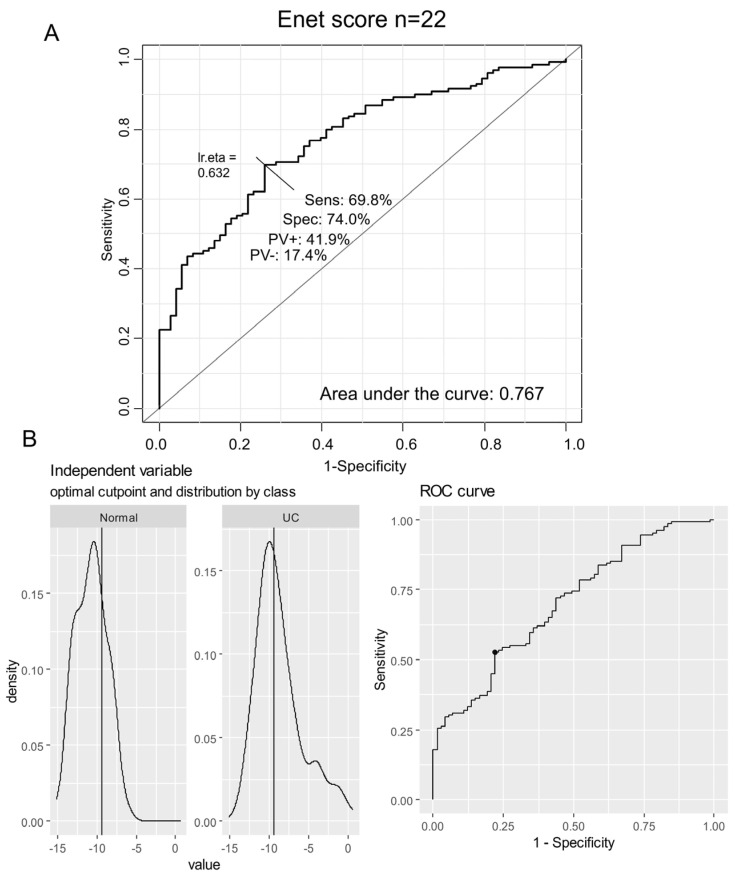
Enet score computation in the validation cohort of ulcerative colitis: GSE11223 dataset. (**A**) ROC curve for combined expression of the 22 activated enzymes in UC samples (AUC: area under the curve, Sens: sensibility, Spec: specificity, PV+: positive prevalence, PV−: negative prevalence); (**B**) Determination of the optimal threshold for the enet score according to UC status of samples.

A receiver operating characteristic (ROC) curve analysis was conducted using the combined expression of the 22 metabolic markers that were validated as overexpressed in both independent cohorts of ulcerative colitis (Figure 3). This analysis, applied to the validation transcriptome dataset, yielded an area under the curve (AUC) of 0.767, demonstrating a sensitivity of 69.8% and a specificity of 74% (Figure 3A). With the expression of the 22 enzymes confirmed as overexpressed in the two independent cohorts, an enzyme Elastic Net expression (enet) score was computed for the GSE11223 transcriptome dataset. The optimal threshold for stratifying the validation cohort based on the enet score was identified at a value of −9.37 (Figure 3B).

**Figure 4:**
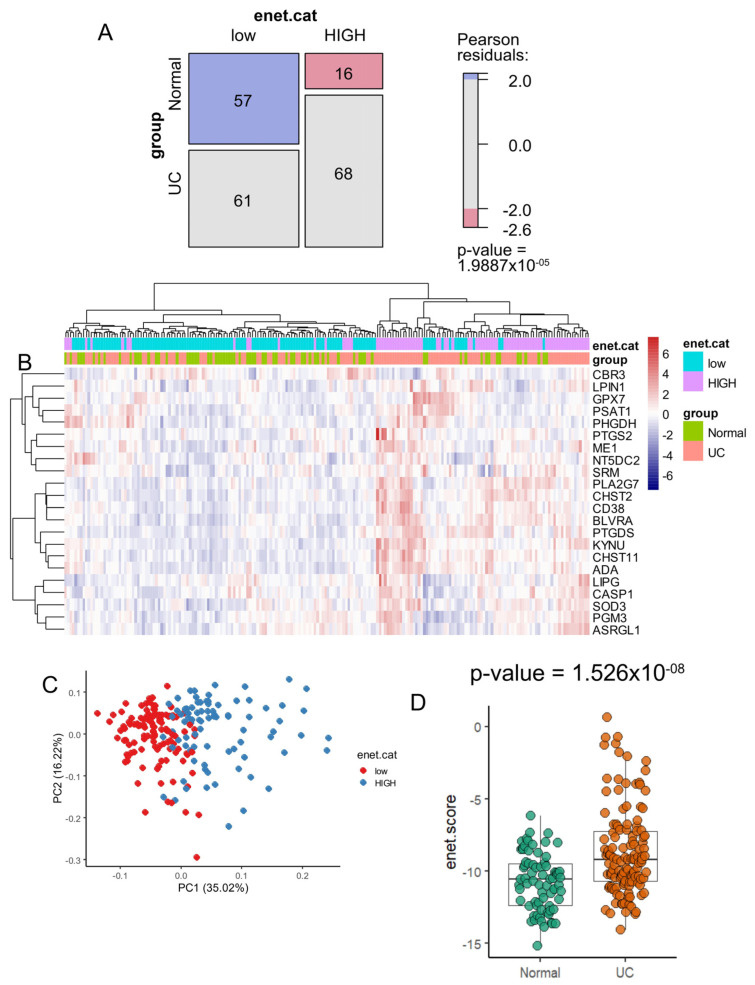
Ulcerative colitis samples harbored higher values of enet score compared to control samples: GSE11223 dataset. (**A**) Mosaic plot and chi-square test between enet score categories (low and high) versus ulcerative colitis (UC) status of samples; (**B**) Principal component analysis based on the expression of the 22 enet activated enzymes; (**C**) Unsupervised clustering (Euclidean distances) and expression heatmap based on the expression of the 22 upregulated enet enzymes; (**D**) Box plot of enet score stratified by group samples: ulcerative colitis (UC) versus normal samples; the *p*-value was evaluated using a two-sided Student’s *t*-test.

A significant difference was observed between the enet score categories (low and high values) concerning ulcerative colitis status, with a chi-square *p*-value of 1.99 × 10^−5^ (Figure 4). This analysis revealed a notable increase in the proportion of samples with a low enet score within the normal control group (Figure 4A). Unsupervised clustering based on the expression of these 22 regulated enzymes allowed for clear stratification of the majority of samples according to their ulcerative colitis status, as well as enet score categories (low and high) (Figure 4B). The expression of the 22 activated enzymes effectively stratified the enet categories in the validation cohort, as demonstrated by an unsupervised principal component analysis (Figure 4C). Additionally, the majority of these 22 enzymes were individually confirmed to be significantly overexpressed in ulcerative colitis samples compared to controls by box plot and two-sided Student’s *t*-test (Supplemental Figure S2). A significantly higher enet score was confirmed in ulcerative colitis samples compared to control samples, as assessed by a two-sided Student’s *t*-test (*p*-value = 1.53 × 10^−8^) (Figure 4D).

The GSE11223 dataset comprised 129 samples of ulcerative colitis disease (Table 2). When this cohort was stratified on metabolism score categories (high score versus low score), a significant difference was observed for tissue sampling, with a higher proportion of inflamed sigmoid colon in the high score category compared to the low score category (*p*-value = 0.03). There was a lower proportion of family history in the high score category (*p*-value = 0.0495199), a higher value of the colonoscopic index of severity in the high score category (*p*-value = 0.0001998), and a lower proportion of disease remission in the high score category (*p*-value = 0.0492499). In summary, in this cohort, patients with samples that harbored higher metabolism score values in their transcriptome seem to be more severe.

### 3.4. Heterogeneity Metabolic of the 22 Enet Upregulated Enzyme During Ulcerative Colitis

**Figure 5:**
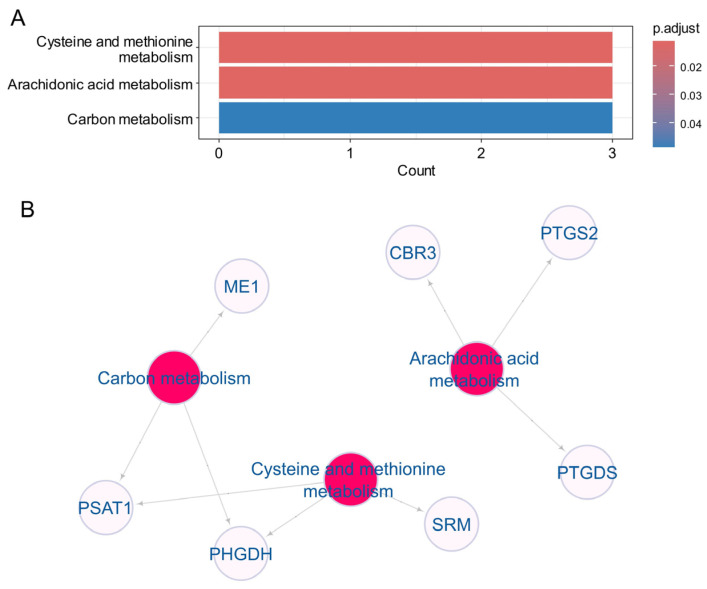
Kyoto Encyclopedia of Genes and Genomes (KEGG) enrichment of the 22 enet activated enzyme during ulcerative colitis: (**A**) Bar plot of KEGG enriched on the 22 enet upregulated enzymes; (**B**) Functional enrichment KEGG network built with the 22 enet upregulated enzymes.

The upregulation of the 22 enet enzymes was confirmed in two independent transcriptome cohorts of ulcerative colitis (GSE38713 and GSE11223), prompting an investigation into their functional enrichment using the Kyoto Encyclopedia of Genes and Genomes (KEGG) metabolic database (Figure 5). This enrichment analysis highlighted the involvement of three key metabolic pathways: cysteine and methionine metabolism, arachidonic acid metabolism, and carbon metabolism (Figure 5A). Specifically, cysteine and methionine metabolism was associated with the enzymes PSAT1, PHGDH, and SRM (Figure 5B), which primarily function in L-serine metabolism and interact with glycolysis (Supplemental Figure S1A). Arachidonic acid metabolism involved the enzymes CBR3, PTGDS, and PTGS2 (Figure 5B), which act downstream of arachidonic acid to produce prostaglandins (Supplemental Figure S1B). Additionally, carbon metabolism was linked to ME1, PSAT1, and PHGDH (Figure 5B).

## 4. Discussion

In this study, we identified 22 upregulated genes encoding enzymes in ulcerative colitis (UC) patients through scRNA-seq analysis. These findings provide important insights into the molecular mechanisms driving UC pathology, particularly highlighting processes such as metabolic reprogramming, immune activation, oxidative stress, and inflammation. A detailed examination of these enzymes reveals their potential roles in UC progression and offers a foundation for considering their clinical relevance as biomarkers or therapeutic targets.

Among the upregulated enzymes, several are involved in metabolic pathways that are key to the behavior of immune cells during inflammation. Notably, LIPG (endothelial lipase) regulates lipid metabolism, a process increasingly recognized for its role in modulating immune responses [42]. Dysregulated lipid metabolism is common in UC and can exacerbate inflammatory signaling. Similarly, PSAT1 and PHGDH, which are key components of the serine biosynthesis pathway, suggest enhanced metabolic activity in immune cells [43]. Increased serine biosynthesis is characteristic of proliferative cells, including those involved in chronic inflammatory conditions like UC [44]. The upregulation of these metabolic enzymes highlights the adaptive metabolic changes occurring in the inflamed tissue, suggesting that targeting metabolic pathways could help modulate immune activation and reduce inflammation.

In parallel, immune regulation and inflammatory signaling were prominently reflected in the upregulation of CD38, CASP1, and PTGS2. CD38 is known for its role in modulating immune responses and regulating cytokine release, which is critical in maintaining the heightened immune activity seen in UC [45,46,47]. CASP1, a key enzyme in the inflammasome pathway, facilitates the production of IL-1β, a pro-inflammatory cytokine central to UC pathology. This indicates that inflammasome activation contributes significantly to the inflammatory cascade in UC, aligning with previous research implicating inflammasome-related pathways in chronic gut inflammation. Similarly, the elevated expression of PTGS2 (also known as COX-2) suggests enhanced prostaglandin synthesis, which is known to drive inflammation [47]. These findings reinforce the notion that inflammatory enzymes such as COX-2 play a central role in UC and may represent viable therapeutic targets; however, the use of COX-2 inhibitors must be approached with caution due to potential gastrointestinal side effects.

Another important aspect of UC pathogenesis is oxidative stress, which is reflected by the upregulation of GPX7 and SOD3. These antioxidant enzymes play a critical role in protecting tissues from the damage caused by reactive oxygen species (ROS) [48,49]. The elevated expression of these enzymes in UC suggests a compensatory response to the increased oxidative stress within the inflamed intestinal tissue. Chronic inflammation in UC is known to generate excess ROS, leading to tissue damage and exacerbation of disease symptoms [50]. By upregulating enzymes that mitigate oxidative stress, the body attempts to limit tissue damage. This points to the potential for antioxidant therapies as a strategy to protect intestinal tissue and reduce the severity of UC.

The integrity of the intestinal barrier is also a crucial factor in UC, and our analysis showed increased expression of CHST2 and CHST11, enzymes involved in glycosaminoglycan biosynthesis [51,52]. Glycosaminoglycans are essential for maintaining the structure and function of the mucosal barrier, which is often compromised in UC. Dysregulation of these enzymes may contribute to the barrier dysfunction observed in UC patients, leading to increased intestinal permeability and immune cell infiltration. Restoring or protecting the intestinal barrier remains a key therapeutic goal, and the enzymes involved in glycosaminoglycan production could represent novel targets for enhancing mucosal healing.

Beyond these functional roles, several of the upregulated enzymes could serve as potential biomarkers for disease activity and prognosis. CD38, CASP1, and PTGS2, for example, could be used to monitor inflammatory activity and assess the severity of UC, with higher expression levels potentially indicating more active or severe disease. Furthermore, the metabolic enzymes PSAT1 and PHGDH could help stratify patients based on immune cell activation and metabolic state, allowing for a more personalized approach to treatment. Such biomarkers would be valuable in guiding clinical decision-making, especially in terms of choosing appropriate immunosuppressive or anti-inflammatory therapies.

From a therapeutic perspective, these enzymes represent promising targets for intervention. Modulating the activity of CASP1 and the inflammasome could reduce the production of IL-1β and other inflammatory cytokines, potentially dampening the chronic inflammatory response in UC. Inhibitors targeting CD38, which is already being explored in other inflammatory conditions and cancers, could help reduce immune cell activation and inflammation in UC. Additionally, targeting enzymes involved in oxidative stress, such as GPX7 and SOD3, might mitigate tissue damage and promote healing in UC patients. Importantly, metabolic interventions aimed at enzymes like PSAT1 and PHGDH could offer novel strategies to regulate immune cell metabolism, potentially reducing the inflammatory burden.

### Clinical Relevance and Utility of Findings

The clinical implications of the 22-enzyme panel identified in this study are substantial. Our analysis demonstrates that the expression levels of these metabolic enzymes can effectively stratify patients with ulcerative colitis (UC), positioning this metabolic signature as a promising non-invasive diagnostic tool. Notably, the validation cohort achieved an area under the curve (AUC) of 0.79, with a sensitivity of 69.8% and specificity of 74%. These metrics suggest that the 22-enzyme signature can reliably differentiate between UC patients and healthy individuals, thus facilitating earlier diagnosis and intervention.

Moreover, the identification of specific metabolic pathways, such as arachidonic acid metabolism, highlights potential therapeutic targets. For instance, the enzymes involved in this pathway are critical in the synthesis of inflammatory mediators like prostaglandins and leukotrienes, which are known to play pivotal roles in UC pathogenesis. Targeting these metabolic enzymes may not only mitigate inflammation but also restore epithelial barrier function, potentially reducing disease severity and improving patient outcomes.

The integration of this metabolic signature into clinical practice could enable personalized treatment strategies based on individual metabolic profiles. By tailoring interventions that consider the specific metabolic dysregulations present in UC patients, clinicians can optimize therapeutic approaches, leading to more effective management of the disease.

Furthermore, this study sets the stage for future research aimed at validating the 22-enzyme signature in larger, multi-center cohorts. Incorporating proteomics and metabolomics data will enhance our understanding of how these metabolic alterations interact with immune responses in UC, thereby refining diagnostic and therapeutic strategies.

In summary, this study not only advances our understanding of UC’s metabolic dimensions but also underscores the potential for a metabolic approach to improve diagnostic accuracy and therapeutic efficacy in managing this complex condition.

## 5. Conclusions

In conclusion, the upregulation of these 22 enzymes in UC provides a window into the complex pathophysiology of the disease, highlighting critical pathways involved in inflammation, metabolism, and oxidative stress. These findings not only deepen our understanding of UC at the molecular level but also point toward potential clinical applications, including biomarker development and novel therapeutic approaches. Further research is warranted to validate these enzymes as biomarkers and explore their therapeutic utility, ultimately aiming to improve disease management and patient outcomes in UC.

## Figures and Tables

**Table 1:** Clinical description of patients from dataset GSE38713: Clinical parameters were stratified group samples comparison from Figure 1: patients from the UC group versus patients from the control group.

GSE38713	Level	Control (n = 20)	UC (n = 23)	Total (n = 43)	*p*-Value
gender	male	7 (35.0)	7 (30.4)	14 (32.6)	
	female	13 (65.0)	16 (69.6)	29 (67.4)	1.0000
group	control_NI (non-inflammatory)	13 (65.0)	0 (0.0)	13 (30.2)	
	UC_remission	0 (0.0)	8 (34.8)	8 (18.6)	
	UC_active	0 (0.0)	15 (65.2)	15 (34.9)	
	control_UC_Ninv (non-involved)	7 (35.0)	0 (0.0)	7 (16.3)	<1 × 10^−4^
age_years	mean (sd)	41 (10.3)	44.8 (10.4)	43 (10.4)	0.2201
disease_extension	Left.sided.colitis	5 (71.4)	14 (60.9)	19 (63.3)	
	Pancolitis	2 (28.6)	9 (39.1)	11 (36.7)	0.9524
	missing	13	0	13	
treatment	Azathioprine	4 (57.1)	11 (47.8)	15 (50.0)	
	5.ASA	3 (42.9)	9 (39.1)	12 (40.0)	
	No.treatment	0 (0.0)	2 (8.7)	2 (6.7)	
	Systemic.steroids	0 (0.0)	1 (4.3)	1 (3.3)	0.7952
	missing	13	0	13	
evolution_time_years	mean (sd)	7.3 (4.5)	8.6 (7.1)	8.3 (6.5)	0.6439
	missing	13	0	13	

**Table 2:** Clinical characteristics of ulcerative colitis samples in GSE11223 stratified according to their metabolism score categories (low and high).

GSE11223	Level	Low (n = 61)	High (n = 68)	Total (n = 129)	*p*-Value
tissue	UC Inflamed terminal ileum	0 (0.0)	1 (1.5)	1 (0.8)	
	UC Inflamed sigmoid colon	9 (14.8)	23 (33.8)	32 (24.8)	
	UC Inflamed descending colon	7 (11.5)	12 (17.6)	19 (14.7)	
	UC Uninflamed ascending colon	13 (21.3)	8 (11.8)	21 (16.3)	
	UC Uninflamed sigmoid colon	13 (21.3)	12 (17.6)	25 (19.4)	
	UC Uninflamed terminal ileum	1 (1.6)	4 (5.9)	5 (3.9)	
	UC Uninflamed descending colon	10 (16.4)	5 (7.4)	15 (11.6)	
	UC Inflamed ascending colon	8 (13.1)	3 (4.4)	11 (8.5)	0.0350474
age_diagnosis	mean (sd)	36.5 (15)	37.2 (14.6)	36.9 (14.8)	0.8027403
joint_problems	FALSE	61 (100.0)	66 (97.1)	127 (98.4)	
	TRUE	0 (0.0)	2 (2.9)	2 (1.6)	0.5246148
uc_fare_up	TRUE	2 (3.3)	10 (14.7)	12 (9.3)	
	FALSE	59 (96.7)	58 (85.3)	117 (90.7)	0.0539442
family_history	FALSE	51 (83.6)	65 (95.6)	116 (89.9)	
	TRUE	10 (16.4)	3 (4.4)	13 (10.1)	0.0495199
Ucss:colonoscopic index of severity	mean (sd)	2 (1.9)	3.7 (3.3)	2.9 (2.9)	0.0001998
ibd_relative	mean (sd)	0.2 (0.5)	0.1 (0.3)	0.1 (0.4)	0.1188165
progression	NEW	2 (3.3)	9 (13.2)	11 (8.5)	
	FAILURE OF THERAPY	15 (24.6)	22 (32.4)	37 (28.7)	
	DISEASE IN REMISSION	44 (72.1)	37 (54.4)	81 (62.8)	0.0492499
smoking_status	ex	27 (44.3)	24 (35.3)	51 (39.5)	
	unknown	0 (0.0)	3 (4.4)	3 (2.3)	
	never	30 (49.2)	32 (47.1)	62 (48.1)	
	current	4 (6.6)	9 (13.2)	13 (10.1)	0.1871728
smoking_amount	15–24	8 (13.1)	6 (8.8)	14 (10.9)	
	unknown	32 (52.5)	35 (51.5)	67 (51.9)	
	5–14	14 (23.0)	15 (22.1)	29 (22.5)	
	25-over	4 (6.6)	4 (5.9)	8 (6.2)	
	0–4	3 (4.9)	8 (11.8)	11 (8.5)	0.6708940
anatomic_location	terminal ileum	1 (1.6)	5 (7.4)	6 (4.7)	
	sigmoid colon	22 (36.1)	35 (51.5)	57 (44.2)	
	descending colon	17 (27.9)	17 (25.0)	34 (26.4)	
	ascending colon	21 (34.4)	11 (16.2)	32 (24.8)	0.0384038
inflammation_status	Inflamed	24 (39.3)	39 (57.4)	63 (48.8)	
	Uninflamed	37 (60.7)	29 (42.6)	66 (51.2)	0.0619667

## Data Availability

R scripts to perform bioinformatics analyses of this article are available at the following web address: https://github.com/cdesterke/UC_metabolism_scripts (accessed on 23 September 2024).

## Supplementary Materials

The following supporting information can be downloaded at: https://www.mdpi.com/article/10.3390/genes15111412/s1, Figure S1: Pathview for enriched KEGG pathways for the enet.22 signature; Figure S2: Box plots of expression for the twenty-two enzymes used to compute the metabolic score in the validation cohort of transcriptome GSE12233; Table S1: Differentially expressed enzymes found between ulcerative colitis and control tissue during analyses of the training transcriptome cohort GSE38713 (10.6084/m9.figshare.27088645) also available at the following address: https://figshare.com/articles/dataset/Table_S1_differential_expressed_enzymes_in_Ulcerative_Colitis_as_compared_to_normal_controls/27088645?file=49362286 (accessed on 23 September 2024).
