# Supplementary material for: Metabolic Transcriptional Activation in Ulcerative Colitis Identified Through scRNA-seq Analysis
Source: Genes (Basel). 2024 Oct 31;15(11):1412. doi: 10.3390/genes15111412 (PMC11593927; doi:10.3390/genes15111412)
Supplement: Supplementary file 1 [file genes-15-01412-s001.zip › genes-3261991-supplementary.pdf]

genes

MDPI

Article

Metabolic Transcriptional Activation in Ulcerative Colitis Identified through scRNA-seq Analysis

Supplemental data

Supplemental Figure S1: Pathview for enriched KEGG pathways for the enet.22 signature: A/ Pathview of methionine and Cysteine metabolism; B/ Pathview of arachidonic acid metabolism

Supplemental Figure S2: Boxplots of expression for the twenty two enzymes used to compute metabolic score in validation cohort of transcriptome GSE12233: expression are presented with the 2 sided Student test p-value to compare the two groups of samples: control versus uc for Ulcerative colitis

Table S1: Differential expressed enzymes found between ulcerative colitis and control tissue during analyses of training transcriptome cohort GSE38713: logFC: logarithm base 2 Fold Change between Ulecerative colitis and control, AveExpr: Average expression of the gene across samples, P-value: LIMMA (linear model for microarray) raw p-value; Adj-P-Val: False Discovery Rate adjusted p-value of LIMMA model.

Figure S1

A

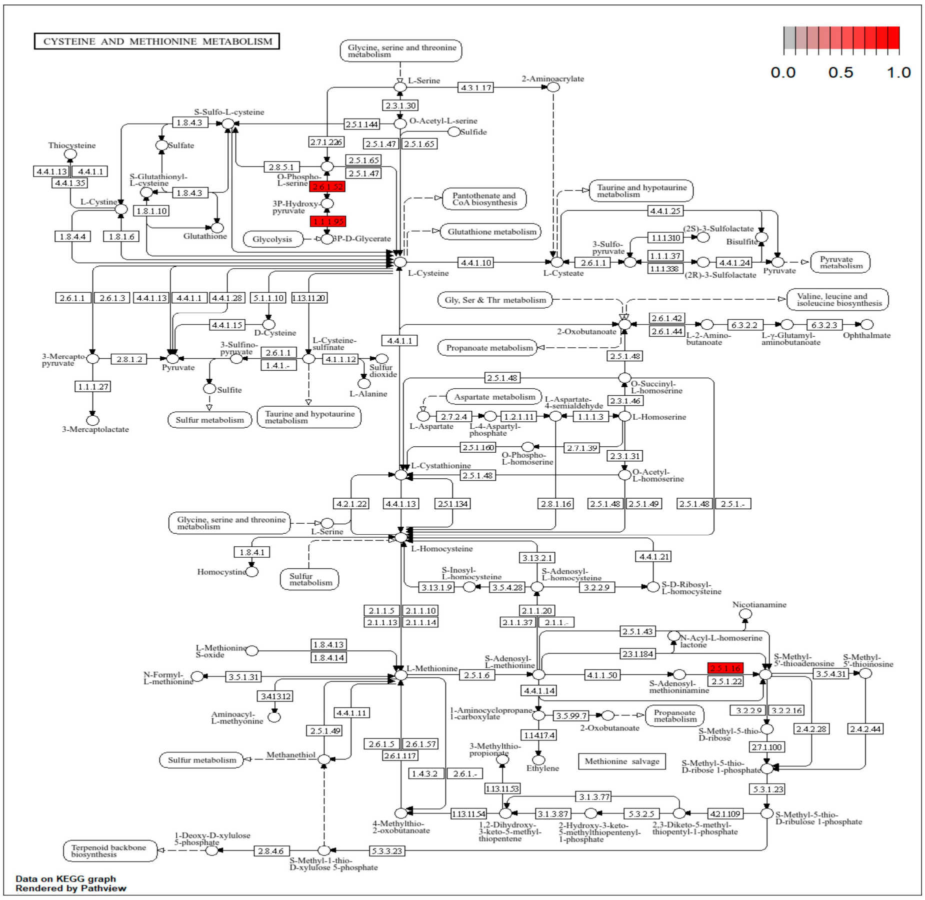
CYSTEINE AND METHIONINE METABOLISM
0.0 0.5 1.0
L-Serine
L-Cysteine
L-Cystathionine
L-Homocysteine
L-Methionine
Methionine salvage
Data on KEGG graph
Rendered by Pathview

B

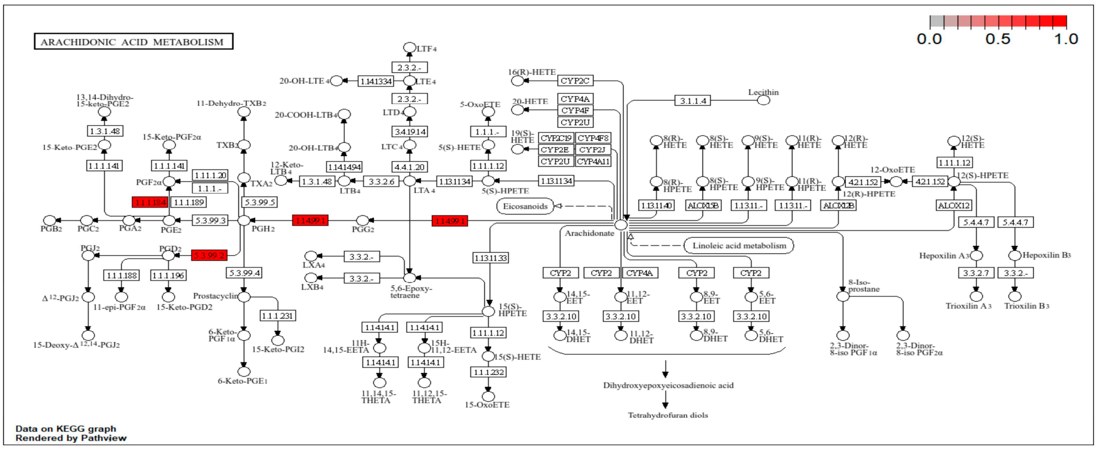
ARACHIDONIC ACID METABOLISM
0.0 0.5 1.0
Arachidonate
Linoleic acid metabolism
Eicosanoids
Dihydroxyepoxyeicosadienoic acid
Tetrahydrofuran diols
Data on KEGG graph
Rendered by Pathview

Figure S2

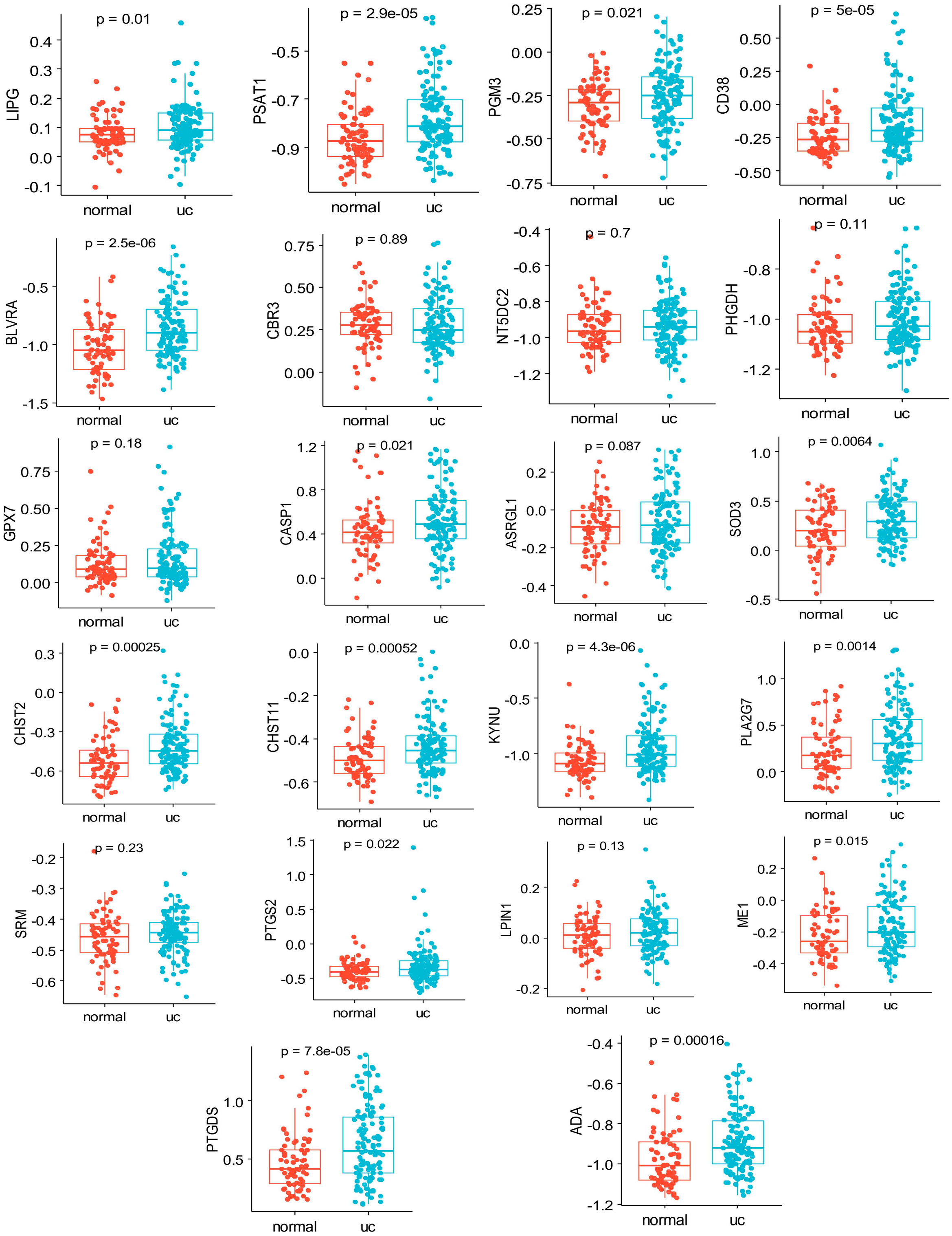
LIPG
p = 0.01
PSAT1
p = 2.9e-05
PGM3
p = 0.021
CD38
p = 5e-05
BLVRA
p = 2.5e-06
CBR3
p = 0.89
NT5DC2
p = 0.7
PHGDH
p = 0.11
GPX7
p = 0.18
CASP1
p = 0.021
ASRGL1
p = 0.087
SOD3
p = 0.0064
CHST2
p = 0.00025
CHST11
p = 0.00052
KYNU
p = 4.3e-06
PLA2G7
p = 0.0014
SRM
p = 0.23
PTGS2
p = 0.022
LPIN1
p = 0.13
ME1
p = 0.015
PTGDS
p = 7.8e-05
ADA
p = 0.00016
normal
uc

Table S1: logFC: logarithm base 2 Fold Change between Ulecerative colitis and control, AveExpr: Average expression of the gene across samples, P-value: LIMMA (linear model for microarray) raw p-value; Adj-P-Val: False Discovery Rate adjusted p-value of LIMMA model.

gene	logFC	AveExpr	P-Value	Adj-P-Val
DDAH2	-1.715	7.948	1.02E-14	1.33E-11
ACADM	-1.209	10.498	4.92E-14	1.64E-11
LPCAT1	3.118	6.900	5.82E-14	1.64E-11
ALDH18A1	-1.065	11.066	6.09E-14	1.64E-11
MTHFD2	1.298	9.523	6.27E-14	1.64E-11
AUH	-1.255	9.426	1.34E-13	2.92E-11
PRDX4	1.124	11.153	2.78E-13	5.20E-11
SEPHS2	-1.246	11.584	4.84E-13	7.93E-11
ACSF2	-2.992	6.349	6.27E-13	9.12E-11
PDK2	-1.999	5.366	7.23E-13	9.46E-11
UGT1A1	-2.266	9.723	1.36E-12	1.62E-10
CYP27A1	-1.877	6.248	1.75E-12	1.86E-10
ME1	2.567	8.432	2.06E-12	1.93E-10
AMACR	-2.503	7.881	2.46E-12	2.01E-10
SRM	1.695	7.398	4.41E-12	2.67E-10
ACAT1	-1.321	10.725	4.57E-12	2.67E-10
LPCAT3	-1.258	6.721	4.57E-12	2.67E-10
PDE8A	-1.018	10.125	4.67E-12	2.67E-10
SUGCT	-1.792	5.728	4.68E-12	2.67E-10
ACOX1	-1.918	9.388	5.93E-12	3.24E-10
MPST	-1.017	9.236	9.70E-12	4.70E-10
FKBP11	2.254	10.510	1.02E-11	4.73E-10
PMM1	-1.426	6.055	1.10E-11	4.81E-10
PLA2G12B	-2.688	4.937	1.15E-11	4.84E-10
ACADS	-1.539	7.541	1.22E-11	4.99E-10
MOCS1	-1.998	4.243	1.98E-11	7.45E-10
ETFDH	-1.483	8.378	2.15E-11	7.83E-10
ADCY9	-1.119	7.819	2.96E-11	1.02E-09
SGMS1	1.239	7.616	3.21E-11	1.08E-09
HSD17B2	-2.151	10.005	4.63E-11	1.52E-09
ACOT8	-1.069	7.405	4.76E-11	1.52E-09
ABAT	-1.975	8.268	5.44E-11	1.69E-09
ASRGL1	1.930	8.544	5.63E-11	1.70E-09
LPIN1	1.896	6.347	5.70E-11	1.70E-09
PKM	1.259	10.022	7.70E-11	2.19E-09
PSAT1	2.538	6.169	8.32E-11	2.32E-09
ALAD	-1.058	8.263	9.14E-11	2.49E-09
BCAT2	-1.085	8.371	1.08E-10	2.82E-09
PHYH	-1.286	10.148	1.42E-10	3.64E-09
CHST2	1.589	6.311	1.84E-10	4.63E-09
PLA2G2A	2.827	12.004	2.21E-10	5.46E-09
RBKS	-1.297	6.740	2.97E-10	6.99E-09
PRDX6	-1.153	12.228	2.99E-10	6.99E-09
PGM3	1.221	8.027	3.06E-10	7.03E-09
CPT1A	-1.135	9.892	4.16E-10	9.38E-09

BDH1	-1.103	8.813	4.65E-10	1.03E-08
HMGCS2	-4.250	10.462	5.34E-10	1.15E-08
HNMT	-1.108	10.814	6.26E-10	1.30E-08
ACSL4	1.667	4.566	7.05E-10	1.42E-08
PDE6A	-1.656	4.569	8.56E-10	1.62E-08
EPHX2	-1.871	7.526	1.06E-09	1.96E-08
DDC	-1.267	10.195	1.14E-09	2.08E-08
CROT	-1.220	7.727	2.15E-09	3.71E-08
ME3	-1.127	6.768	2.74E-09	4.53E-08
AHCYL2	-1.293	10.513	2.76E-09	4.53E-08
ENTPD1	1.763	6.765	2.77E-09	4.53E-08
SDR16C5	2.056	8.814	2.82E-09	4.56E-08
GSTM4	-1.358	7.347	3.06E-09	4.83E-08
CRAT	-1.086	8.626	3.16E-09	4.92E-08
PFKFB3	2.039	7.282	3.36E-09	5.17E-08
NT5DC2	1.501	4.727	4.16E-09	6.18E-08
KYNU	3.801	5.827	5.80E-09	8.25E-08
ENTPD5	-1.799	10.450	7.86E-09	1.07E-07
GPX8	2.362	6.345	1.09E-08	1.45E-07
CTH	-1.074	7.496	1.12E-08	1.47E-07
MAOA	-1.412	11.319	1.15E-08	1.49E-07
ASS1	1.297	12.270	1.24E-08	1.58E-07
CRYL1	-1.072	9.063	1.24E-08	1.58E-07
ADH1A	-1.101	4.275	1.31E-08	1.61E-07
ACADSB	-1.167	7.225	1.64E-08	1.96E-07
LIPG	2.020	5.766	1.69E-08	1.99E-07
PFKP	1.008	9.093	1.74E-08	2.02E-07
CHKA	-1.067	6.317	2.54E-08	2.77E-07
PUS10	-1.032	4.830	4.03E-08	4.15E-07
ENPP2	1.650	7.825	4.42E-08	4.49E-07
SULT1B1	-1.163	8.863	5.12E-08	5.07E-07
SORD	1.173	8.931	5.43E-08	5.34E-07
CES2	-1.149	12.331	5.62E-08	5.47E-07
CLYBL	-1.454	7.739	5.81E-08	5.58E-07
TXNDC15	1.193	9.080	5.88E-08	5.58E-07
HMOX1	-1.517	7.527	6.35E-08	5.89E-07
SRD5A3	2.006	7.804	7.05E-08	6.45E-07
NUDT7	-1.485	6.894	7.46E-08	6.78E-07
TBXAS1	1.214	5.496	8.01E-08	7.17E-07
NAT2	-1.122	8.635	9.55E-08	8.22E-07
CPT2	-1.267	8.598	1.00E-07	8.53E-07
UGT2A3	-2.979	8.630	1.06E-07	8.91E-07
BPNT1	-1.195	7.771	1.10E-07	9.20E-07
CBS	-1.848	3.619	1.19E-07	9.77E-07
ERI1	1.067	6.064	1.42E-07	1.15E-06
CES3	-1.058	8.655	1.54E-07	1.23E-06
LDHD	-1.566	5.457	1.54E-07	1.23E-06
PCK1	-3.791	10.377	1.71E-07	1.34E-06
CHST15	1.976	6.336	2.07E-07	1.56E-06
EHHADH	-1.045	7.441	2.48E-07	1.82E-06
SOD3	1.250	4.973	2.52E-07	1.84E-06
LIAS	-1.514	5.891	3.14E-07	2.21E-06
CBR3	1.496	5.299	3.31E-07	2.30E-06

ATP8A1	-1.165	8.827	3.95E-07	2.72E-06
PHGDH	1.132	3.684	5.04E-07	3.32E-06
CHST11	1.861	4.518	5.40E-07	3.52E-06
NEU1	-1.147	7.567	5.58E-07	3.62E-06
SCD	1.476	9.014	6.48E-07	4.16E-06
ENPP1	-2.030	5.095	9.23E-07	5.75E-06
CD38	2.592	5.582	1.01E-06	6.26E-06
ACSM3	-1.022	7.938	1.06E-06	6.51E-06
ALDH5A1	-1.199	7.000	1.23E-06	7.33E-06
ADA	1.261	3.331	1.31E-06	7.74E-06
AIFM3	-1.579	6.268	1.34E-06	7.86E-06
ALOX5	1.454	7.778	1.71E-06	9.66E-06
ACAA2	-1.055	11.079	1.76E-06	9.83E-06
BCKDHB	-1.028	7.424	1.92E-06	1.05E-05
ADH1C	-2.735	11.049	1.97E-06	1.07E-05
FASN	1.134	5.513	2.14E-06	1.14E-05
CASP1	1.408	10.669	2.41E-06	1.26E-05
PFKFB2	-1.292	7.508	3.04E-06	1.54E-05
SQLE	1.153	8.100	3.42E-06	1.72E-05
BLVRA	1.290	6.308	3.81E-06	1.89E-05
PLA2G12A	-1.179	7.921	4.64E-06	2.24E-05
STEAP4	2.103	4.113	4.92E-06	2.35E-05
FUT8	1.154	7.669	5.17E-06	2.45E-05
HSD3B2	-2.088	3.490	6.17E-06	2.87E-05
PTGS1	1.339	6.236	6.44E-06	2.98E-05
MSRB3	1.622	6.324	7.31E-06	3.30E-05
PLA2G7	1.392	7.439	7.51E-06	3.38E-05
PANK1	-1.441	9.131	7.61E-06	3.41E-05
NOS2	2.738	5.449	7.69E-06	3.44E-05
PDE4B	2.056	6.185	7.81E-06	3.48E-05
LPL	1.062	3.538	1.01E-05	4.41E-05
MOGAT2	-1.248	7.468	1.10E-05	4.75E-05
PLOD2	-1.029	10.534	2.80E-05	1.11E-04
CARD16	1.021	9.995	2.89E-05	1.14E-04
BCAT1	1.279	4.412	3.28E-05	1.27E-04
PTGDS	1.819	7.647	3.76E-05	1.43E-04
PDE3A	-1.080	8.064	3.89E-05	1.47E-04
OTC	-1.263	3.196	4.96E-05	1.81E-04
KMO	1.186	2.874	5.75E-05	2.07E-04
HSD11B2	-1.146	10.348	6.02E-05	2.16E-04
TDO2	2.332	4.424	7.25E-05	2.55E-04
GPX7	1.298	3.008	7.38E-05	2.59E-04
ALDH1A2	2.114	3.259	8.39E-05	2.88E-04
PTGS2	2.030	5.169	5.04E-04	1.50E-03
HSD11B1	1.203	3.014	7.48E-04	2.11E-03
CKB	-1.384	11.454	8.26E-04	2.31E-03
ALDOB	2.229	7.614	9.02E-04	2.48E-03
